# Vitamin C Affinity to TiO_2_ Nanotubes: A Computational Study by Hybrid Density Functional Theory Calculations

**DOI:** 10.3390/nano14030261

**Published:** 2024-01-25

**Authors:** Aldo Ugolotti, Mirko Dolce, Cristiana Di Valentin

**Affiliations:** 1 Dipartimento di Scienza dei Materiali, Università di Milano-Bicocca, via R. Cozzi 55, 20125 Milano, Italy; aldo.ugolotti@unimib.it (A.U.);; 2 BioNanoMedicine Center NANOMIB, Università di Milano-Bicocca, via Follereau 3, 20854 Vedano al Lambro, Italy

**Keywords:** TiO_2_, titania nanotubes, vitamin C, drug transport, DFT, drug adsorption

## Abstract

Titanium dioxide nanotubes (TNT) have been extensively studied because of their unique properties, which make such systems ideal candidates for biomedical application, especially for the targeted release of drugs. However, knowledge about the properties of TiO_2_ nanotubes with typical dimensions of the order of the nanometer is limited, especially concerning the adsorption of molecules that can be potentially loaded in actual devices. In this work, we investigate, by means of simulations based on hybrid density functional theory, the adsorption of Vitamin C molecules on different nanotubes through a comparative analysis of the properties of different structures. We consider two different anatase TiO_2_ surfaces, the most stable (101) and the more reactive (001)A; we evaluate the role of the curvature, the thickness and of the diameter as well as of the rolling direction of the nanotube. Different orientations of the molecule with respect to the surface are studied in order to identify any trends in the adsorption mechanism. Our results show that there is no preferential functional group of the molecule interacting with the substrate, nor any definite spatial dependency, like a rolling orientation or the concavity of the nanotube. Instead, the adsorption is driven by geometrical factors only, i.e., the favorable matching of the position and the alignment of any functional groups with undercoordinated Ti atoms of the surface, through the interplay between chemical and hydrogen bonds. Differently from flat slabs, thicker nanotubes do not improve the stability of the adsorption, but rather develop weaker interactions, due to the enhanced curvature of the substrate layers.

## 1. Introduction

The discovery of carbon nanotubes in 1991 was accompanied by the rapid and continuous development of the research field on one-dimensional (1D) materials. Particular attention has been paid in understanding the relationship between the dimensionality of the material and its physico-chemical properties. The Single-Walled Carbon Nanotube (SWCNT) is the progenitor of 1D materials and is characterized by a single layer of sp^2^ hybridized carbon atoms rolled up to form a cylindrical structure. The extensive surface area, the π conjugation inside and outside the nanotube and the consequent high thermal and electrical conductivity are properties that are useful for a variety of potential technological applications [1].

In recent years, titania nanotubes (TNTs) have attracted much attention, because they are expected to combine the unique characteristics of CNTs, both in terms of their versatility and of the possibility to modulate length, radius and thickness [2,3], with the reactivity of TiO_2_ nanostructures, which have extensive applications ranging from photocatalysis and photoelectrochemistry for environmental and energy processes [4,5]. In addition, due to their high availability in nature, non-toxicity, corrosion resistance and biocompatibility, they are also largely used in cosmesis and, more recently, in biomedical applications. TNTs are particularly useful as drug-releasing implants [6].

Many chemical and physical preparation techniques are known for obtaining titania nanotubes. Among the most important ones are sol–gel techniques, methods based on the use of templating agents, hydro/solvo-thermal approaches and electrochemical anodization [7,8,9,10]. More ordered arrays of nanotubes with larger diameters (of the order of 50–200 nm) can be obtained through electrochemical anodization, which is, thus, the most used technique, especially for biomedical applications, as it allows greater control of the growth parameters. For instance, it is possible to grow nanotubes with a specific preferential exposed surface [11,12,13].

The investigation of the morphology and the structural characterization of nanotubes can be carried out through multiple techniques. For an atomic level of detail, Scanning Tunneling Microscopy (STM) or Transmission Electronic Microscopy (TEM) have been used [14], whereas through X-ray photoelectron spectroscopy (XPS), the chemical structure of nanotubes was determined [15]. X-ray or neutron diffraction and infrared or Raman spectroscopy provide information on the degree of crystallinity and on crystalline phases [16]. However, details on the exposed surface and on how the nanotubes are rolled up are almost never present. Given this gap in the literature, the characterization of titania nanotubes by means of theoretical modeling could be very useful to the community working in the field.

Titania nanotubes have the excellent ability to adsorb multiple atomic or molecular species [17,18], which is particularly exploited for biomedical applications. Often, these materials are incorporated into metal implants for the administration of precise quantities of a drug through its controlled release, which is an efficient approach to avoid overdose and the related side effects. The drug release can be induced by applying external stimuli, including pH variation, light, thermal, electrical or magnetic stimuli [19,20]. The drugs most commonly considered in the literature are anti-inflammatories and antibiotics [21,22], such as ibuprofen [23,24], quercetin [25], propolis [26] and the anticancer drug doxorubicin [27]. They can serve both as therapeutic agents and to reduce the impact of the implant on the surrounding tissue, improving cell proliferation and osseointegration. 

**Figure 1:**
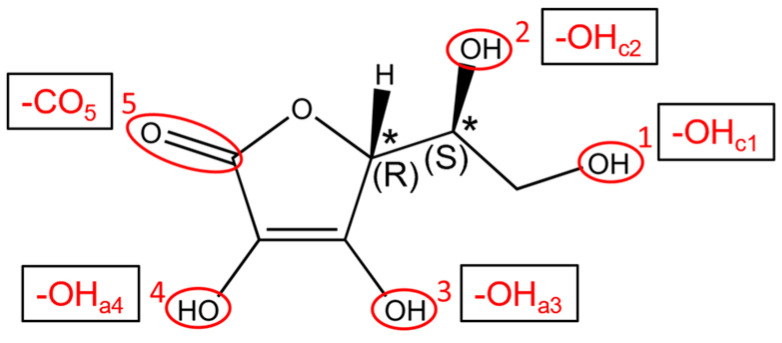
Chemical structure of Vitamin C and details about the configuration of stereogenic centers. The five anchoring groups are numbered and labeled. The * labels mark chiral centers.

In the literature, there are only a few examples where the ability of TNTs to carry and release drugs has been investigated through a combined experimental and computational study, such as in Refs. [28,29]. The bioactive molecular species under investigation here is *L*-ascorbic acid (Vitamin C), sketched in Figure 1, which is characterized by excellent antioxidant properties.

The aim of the present work is to investigate, by means of first-principles calculations, the adsorption of Vitamin C molecules on different TNTs and to find a rationale to their use for the transport of drugs. Therefore, we carried out a comparative analysis between different characteristics of the nanotubes that were expected to drive their chemical properties at the nanoscale: exposed surface, diameter, thickness and rolling orientation. We considered both the most stable anatase (101) surface [30,31,32] and the more reactive (001)A one, which is the only one that has been reported to spontaneously roll up [33,34,35,36]. We built TNT models with diameters of ca. 27–105 Å, with one or three Ti layers, and we identified trends in the adsorption configuration. 

## 2. Computational Details

We carried out the geometry optimizations and the electronic structure calculations within the density functional theory (DFT) framework, through the CRYSTAL17 software [37,38]. We expanded the Kohn–Sham orbitals using Gaussian-type orbitals, with an all-electron basis set as (Ti) 86-4111 (d41) and (O) 8-4111 (d1) for TiO_2_ and (H) 5-111 (p1), (C) 6-311(d1) and (O) 8-41111 (d1) for the Vitamin C molecule [39]. We used the B3LYP hybrid functional [40,41], and we included the dispersion interaction through Grimme’s D* correction [42,43]. For the calculation of the Vitamin C adsorption on the flat anatase TiO_2_ (101) surface, we used a supercell built from the 1 × 4 repetition of the conventional cell, where only one molecule was included to minimize interactions between periodic replicas. The asymmetric layer of the slab includes two O atoms per single Ti, in line with the stoichiometry of the system. In the case of the thicker slab model, we included three asymmetric triatomic layers (which we labeled 3L), and we fixed the supercell lattice parameters to those derived from the optimized anatase bulk with a = 3.76 Å and c = 9.76 Å (expt. 3.78 Å and 9.50 Å, respectively [44]). Then, we relaxed the coordinates of the first two triatomic layers (and those of the molecule), keeping the third one fixed to simulate the constraint introduced by thicker supports. In contrast, we modeled thinner slabs by including a single asymmetric layer (which we labeled 1L) and we performed the full optimization of both supercell axes and atomic coordinates.

In the case of slabs based on the (001) surface, we optimized a 4 × 3 repetition of the surface’s unit cell in order to maintain a coverage similar to that obtained for the (101) surface. We fixed the supercell structure to that derived from the bulk, with supercell a′ = b′ = 3.41 Å. The (001)A surface has been reported to be a more stable reconstruction of the conventional (001) one, differing by a translation on the second and third Ti layers along the a’ and b’ directions, respectively; a sketch of the structure of the different (001) surfaces is shown in Figure S1 in the Supplementary Materials. For both the slab and the nanotubes built on the (001)A surface, we only considered the case of thick supports, i.e., with three layers, because thinner layers would not represent crystal cut surfaces.

For all the slabs, the convergence on the electronic structure during the optimization was achieved using a reciprocal space grid of 2 × 2 × 1 k-points, which was increased to 16 × 16 × 1 for the calculation of the density of states (DOS).

The structure of the nanotubes was completely defined through the rolling vector R = (*n*, *m*), where the *n*, *m* indexes are multipliers of the crystal axes of the conventional unit cell of the surface [45]. We want to highlight that, if an *l* × *j* supercell is taken as a starting point of the rolling procedure, in order to keep the diameter of a TNT constant, the rolling vector has to be re-scaled accordingly, for example, by using R = (*n/l*, *m*) when rolling along the x of the supercell. However, we will refer to the construction of a TNT based on the conventional unit cell. 

In the case of thin (1L) TNTs exposing the (101) anatase TiO_2_ surface, we built our models starting from both R = (0, 24) and R = (8, 0), representing the [10-1] and [010] rolling directions, respectively, with a diameter of c.a. 27 Å. For these structures, we considered the adsorption of Vitamin C molecules both outside and inside the TNT: in the first case, we rolled up a 1 × 4 slab supercell, through C_6_ or C_8_ symmetries (i.e., R = (0, 6) or R = (8, 0), respectively), depending on the rolling direction. In the second case, we had to roll up a larger supercell, i.e., 1 × 8 or 2 × 4, respectively, in order to minimize the interaction between a molecule and its symmetrical replicas.

In the case of the thicker 3L models based on the (101) or the (001)A surface, we noticed that, because the diameter was too small, the outer layers exhibited Ti-O distances that were too long, therefore being unrealistic (as shown in Figures S2 and S3). Therefore, we considered the minimum diameter which provided a more accurate chemical description of the TNT, corresponding to c.a. 105 Å and 34 Å for the (0, 96) (101) nanotube and the (36, 0) (001)A one, respectively.

For all the TNT models, the only periodic direction of the system corresponded to the axis of the nanotube. Both the coordinates of the atoms and the lattice parameter along the TNT axis were left free to relax; the optimization and the calculation of the DOS were performed at the Γ point only.

Since the goal of our investigation was to identify trends in the adsorption of Vitamin C on TiO_2_ slabs or nanotubes, in order to keep the computational requirements affordable even for the largest slabs and nanotubes, we did not account explicitly for the basis set superposition error (BSSE). However, to provide a basic assessment of the impact of the overlap of the basis set functions on our results, we calculated a posteriori the BSSE correction for the most stable adsorption mode on the (101) anatase TiO_2_ surface, i.e., the B^1Hbond-A^_O(H)a3/OHa4_ model (see below), for both the 3L and 1L slabs. We relied on the geometrical counterpoise correction (gCP) method [46,47], for which $E_{BSSE}=E^{gCP}(\mathrm{Vit}C/TiO_{2})-E^{gCP}(TiO_{2})-E^{gCP}\left(\mathrm{Vit}C\right)$, with semi-empirical parameters taken from Ref. [46]. For the two independent TiO_2_ slab and Vitamin C molecule structures, we took the geometry already optimized for the B^1Hbond-A^_O(H)a3/OHa4_ model. Since for both the 1L and 3L slabs we calculated E_BSSE_ = 0.7 eV, we assumed that a similar correction of the order of a few tenths of eV should be applied to all our calculated chemisorption energies, reducing their magnitudes. For the two physisorbed models of large 3L nanotubes, the calculated E_BSSE_ was smaller, i.e., 0.5 eV.

## 3. Results and Discussion

### 3.1. Vitamin C Adsorbed on the Anatase (101) TiO_2_ Surface and the Corresponding Nanotubes

We started our investigation of the adsorption of Vitamin C molecules on anatase TiO_2_ surfaces by carrying out a survey of different adsorption configurations on both the fully optimized 1L and the crystal-cut 3L slab, considering the five different functional groups that can interact with the surface, which we labeled OH_c1_, OH_c2_, OH_a3_, OH_a4_ and CO_5_, reported in Figure 1. The most promising models were then taken into consideration when moving from flat slab surface models to curved nanotube ones.

The optimized configurations were then labeled as X^Z^_Y_, where X = (M, B) corresponds to Vitamin C adsorbed through one (monodentate, M) or two (bidentate, B) functional groups and Y indicates the functional groups interacting with the surface. The superscript Z is in the form “*n* Hbonds-A/D” and reports the formation of *n* hydrogen bonds and their proton acceptor/donor (A/D) behavior. In the case of hydroxyl groups, the eventual deprotonation is marked by putting a bracket around the H, for example, as O(H)_a3_. 

**Figure 2:**
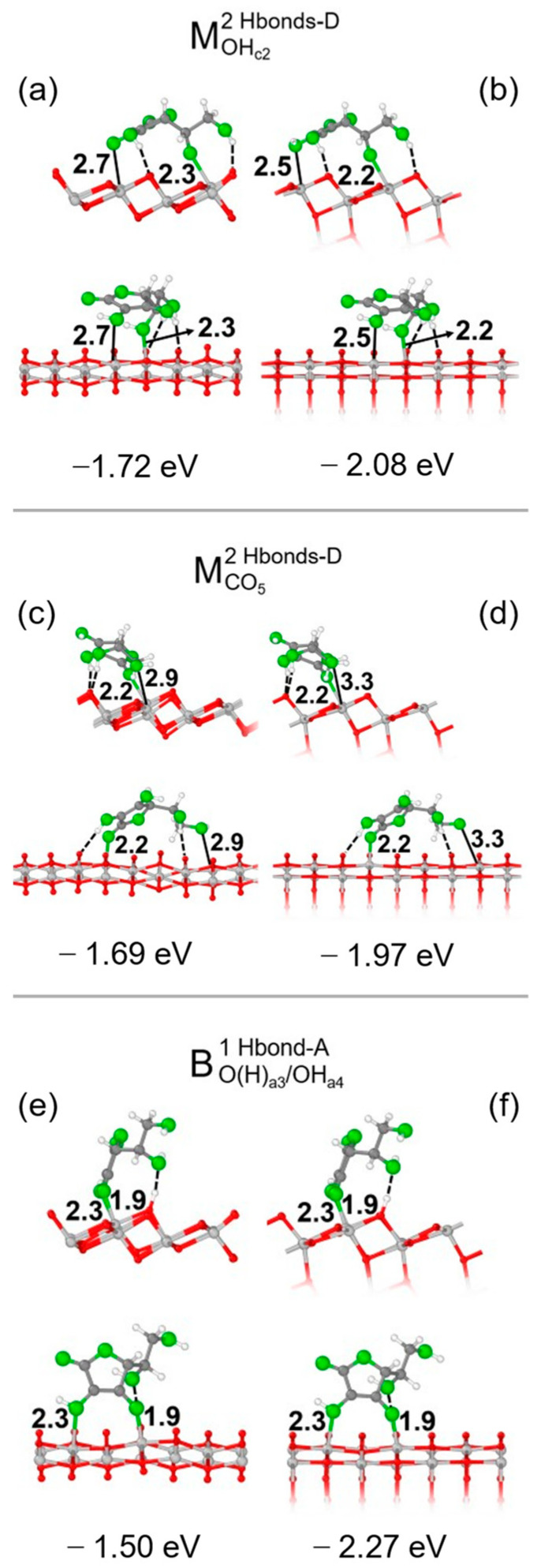
Adsorption configurations and energies per molecule (${\Delta E}_{\mathrm{ads}}^{\mathrm{mol}}$) for three optimized models of Vitamin C on the TiO_2_ (101) surface: $M_{OHc2}^{2Hbonds-D}$, $M_{CO5}^{2Hbonds-D}$ and $B_{O(H)a3/OHa4}^{1Hbond-A}$. In each panel, the top/bottom image reports the front/side view along the [010]/[10-1] crystallographic direction. Panels (**a**,**c**,**e**) show the 1L slab, while panels (**b**,**d**,**f**) show the 3L slab. Ti, C and H atoms are represented with light gray, dark gray and white spheres, respectively. Oxygen atoms of Vitamin C/slab are represented with green/red spheres. Solid and dashed black lines mark the electrostatic and hydrogen bonds, respectively. Relevant distances are reported in Å.

For each structure, we calculated the adsorption energy per molecule, defined as ΔE^mol^_ads_ = E_slab + mol_ − E_slab_ − E_mol_, and focused on the three most stable adsorption configurations, i.e., M^2Hbonds-D^_OHc2_, M^2Hbonds-D^_CO5_ and B^1Hbond-A^_O(H)a3/OHa4_, which are shown in Figure 2 for both the 1L and 3L slab. Additional details on other models we investigated are shown in Figure S4. The average lattice parameters a’, b’ of the 1L primitive cell of the TiO_2_ slab optimized in the presence of Vitamin C were slightly different than those of the bulk: on average, they were 3.50 Å, 10.50 Å vs. 3.76 Å, 10.46 Å. Generally speaking, an interplay due to the formation of chemical and/or hydrogen bonds could be observed. In the monodentate configurations, only one functional group of the molecule bonds with an undercoordinated Ti atom on the surface compared with two O-Ti bonds of the bidentate structure; however, in the latter model, only one H-bond was formed, even though the adsorption energies of the three structures were rather similar. 

Since there are so many interaction possibilities between the functional groups of Vitamin C and the substrate, the dispersion contribution given by the C ring of the molecule, which is crucial in the case of catechol and dopamine on the same surface [39], did not determine the most stable structure here. 

It is crucial to note that the adsorption energies and geometries of the 3L slab were similar to those of the 1L one (see data for ΔE^mol^_ads_ in Figure 2); the additional layers contributed to stabilizing the adsorption structure without significantly changing the overall geometry. Therefore, the adsorption behavior was similar for thin slabs, which were the main target of our analysis, and thick slabs, which were the prototypes of realistic TNTs.

**Figure 3:**
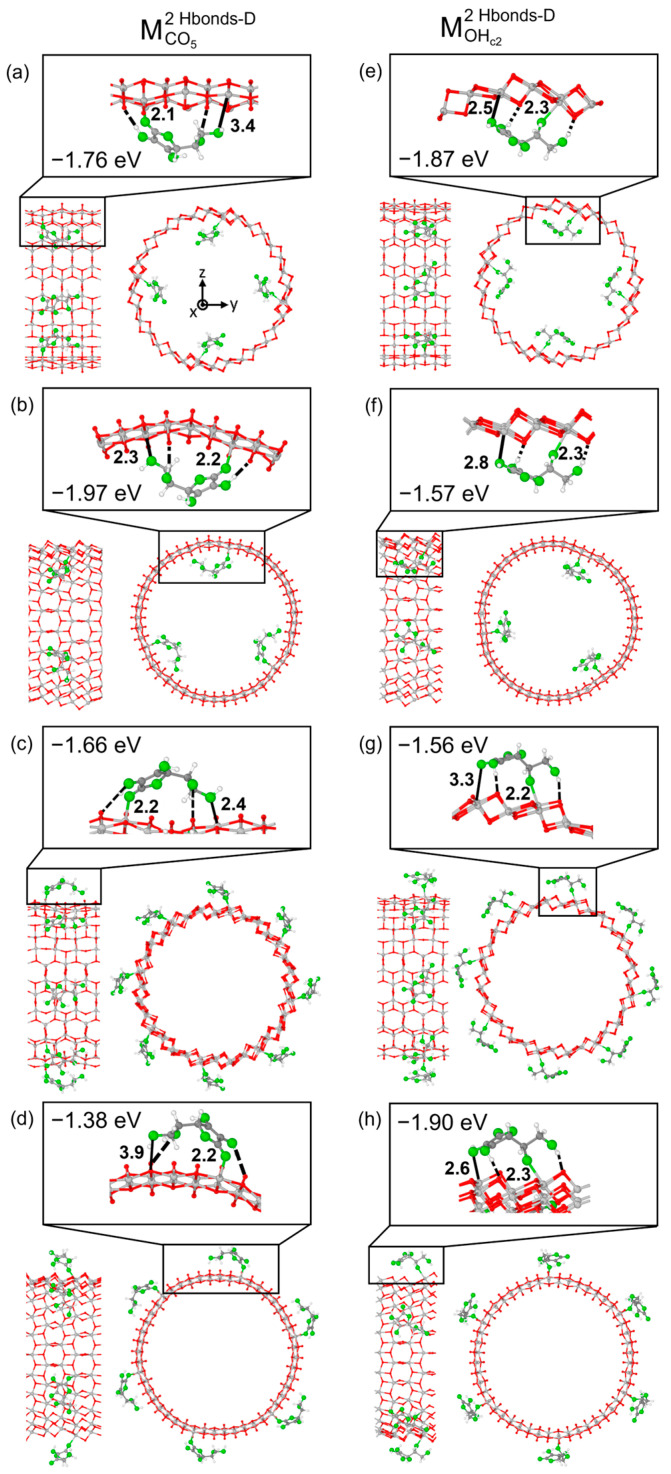
Optimized configurations and adsorption energies per molecule (${\Delta E}_{\mathrm{ads}}^{\mathrm{mol}}$) for two models of Vitamin C: $M_{CO5}^{2Hbonds-D}$ and $M_{OHc2}^{2Hbonds-D}$ adsorbed on different TiO_2_ NT (101) 1L configurations. In panels (**a**,**b**,**e**,**f**), Vitamin C is placed inside the NT, while in panels (**c**,**g**,**d**,**h**), Vitamin C is placed outside the NT. In panels (**a**,**c**,**e**,**g**), the NT is constructed with a (0, 24) roll-up index, while in panels (**b**,**d**,**f**,**h**), with a (24, 0) index. In each panel, the left/right image reports the front/side view, while on top, a zoom-in on the molecule is shown. Ti, C and H atoms are represented with light gray, dark gray and white spheres, respectively. Oxygen atoms of Vitamin C/slab are represented with green/red spheres. Solid and dashed black lines mark the electrostatic and hydrogen bonds, respectively. Relevant distances are reported in Å.

We now move to TNT and first report on the interactions between Vitamin C molecules and TiO_2_ nanotubes exposing the anatase (101) surface. Among the most stable adsorption modes discussed above for the slab models, we focus on the two monodentate configurations, because they should be less dependent on the orientation of the molecule with respect to the surface, which allows us to evaluate all degrees of freedom when adsorbing on TNTs, for example, along the TNT rolling directions. As reported in Figure 3, we considered Vitamin C placed both inside or outside the nanotube and rolling the surface along either in the [010] or the [10-1] direction. To explain the limitation encountered with bidentate adsorption modes, we mention that by building up the adsorption models by rolling the B^1Hbond-A^_O(H)a3/OHa4_ slab configuration, with the molecule placed inside the TNT, the Vitamin C was largely distorted, therefore experiencing unrealistic compression. We show its structure in Figure S5 for reference only. 

Despite minimal variations in the adsorption geometries, several observations can be made from the comparison of the adsorption energies between the different models. In the case of structures based on the M^2Hbonds-D^_CO5_ slab configuration, if the long axis of the molecule was aligned to the nanotube axis (Figure 3a,c), the adsorption energy changed only slightly from that of the 1L slab, from −1.69 eV to −1.76 eV/−1.66 eV, depending on whether Vitamin C was inside or outside the TNT. A larger variation in the adsorption energy was observed (−1.97 eV/inside and −1.38 eV/outside), as expected, when the long axis of the molecule was aligned to the rolling direction of the nanotube (Figure 3b,d). Differently, these trends could not be identified in the case of the structures based on the M^2Hbonds-D^_OHc2_ slab configuration. Here, the strength of the adsorption was correlated with the bond length between the undercoordinated Ti atom at a surface and the O of one of the hydroxyl groups of the molecule. For example, in M^2Hbonds-D^_OHc2_ TNT models, shorter Ti- OH_a3_ distances (Figure 3e,g) with respect to the slab structure (2.5 Å /2.6 Å vs. 2.7 Å) corresponded to an increase in the adsorption energy (−1.87 eV/−1.90 eV vs. −1.72 eV). 

**Figure 4:**
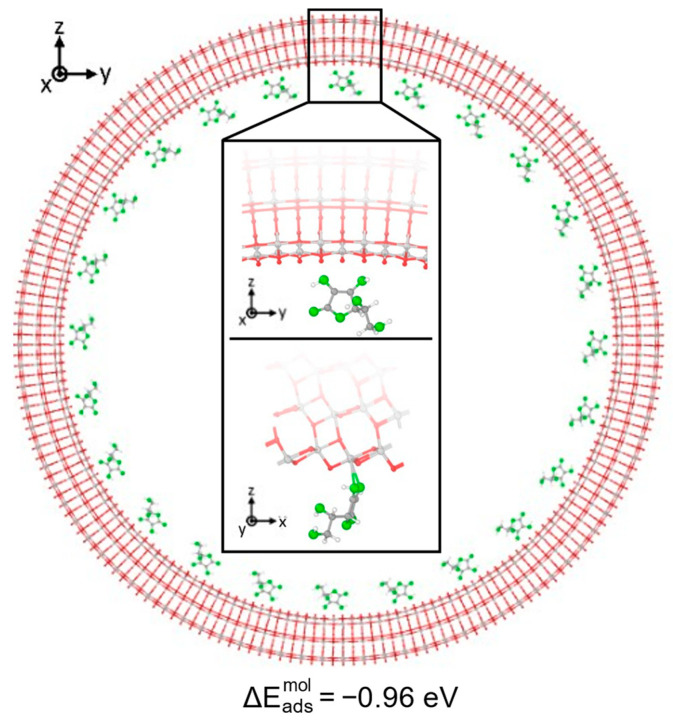
Adsorption configuration and energy for Vitamin C placed inside the TiO_2_ NT (101) 3L (96, 0). The larger image shows a longitudinal slice of the structure, while in the inset, the top/bottom image shows a side/front view of a zoom-in on the Vitamin C. Ti, C, H atoms are represented with light gray, dark gray and white spheres, respectively. Oxygen atoms of Vitamin C/slab are represented with green/red spheres. Solid and dashed black lines mark the electrostatic and hydrogen bonds, respectively. Relevant distances are reported in Å.

We now move from a thin to a thick and quite large 3L (96, 0) TNT, as shown in Figure 4, to reach more realistic conditions. In this case, we only placed the Vitamin C molecules inside the nanotube, in order to mimic the drug loaded in an actual implant, and we optimized our model starting from the most stable configuration on the 3L slab surface, which is the B^1Hbond-A^_O(H)a3/OHa4_ configuration. We must notice that here, since the radius of the 3L (96, 0) nanotube was 4 times larger than that for the 1L nanotubes, we did not observe any unrealistic compression for bidentate structures as before. Interestingly, despite the increased thickness, which was expected to strongly stabilize the adsorption, as observed from 1L to 3L slabs, the overall interaction on the 3L (96, 0) TNT was weaker than all the previous cases considered, since the adsorption energy was only −0.96 eV. Observing the optimized structure, indeed, both the OH-Ti bonds were elongated with respect to the B^1Hbond-A^_O(H)a3/OHa4_ 3L slab configuration, 2.4 Å vs. 2.3 Å, and no H-bonds were formed. In addition, none of the hydroxyl groups of the C ring were found to favorably become deprotonated, despite the relatively low value of the acid dissociation constant for Vitamin C (pKa = 4.17) [48]. This result is in contrast with the bidentate configuration on both the 1L and the 3L slabs, where a proton was favorably transferred to a nearby bridging O of the surface, which formed an additional H-bond with the hydroxyl group in the side chain of the Vitamin C, further stabilizing the adsorption. The deprotonation of similar molecules, like formic acid [49] or catechol/dopamine [39], was already reported on the anatase (101) TiO_2_ surface; therefore, we attribute the different behavior to the curvature of the support. Therefore, we deem the interaction between the Vitamin C and the anatase (101) TiO_2_ surface to be driven by geometrical factors, i.e., the correspondence and the correct alignment of any functional groups of the molecule with the chemically active undercoordinated Ti atoms or bridging O (for the formation of H-bonds).

In the following, we will investigate how the electronic properties of the bare nanotube and of the Vitamin C change upon adsorption. We focus our efforts on the case of adsorption models on both 1L and 3L slabs, which are our reference configurations, and on the most stable adsorption modes on the 1L TNT. 

**Figure 5:**
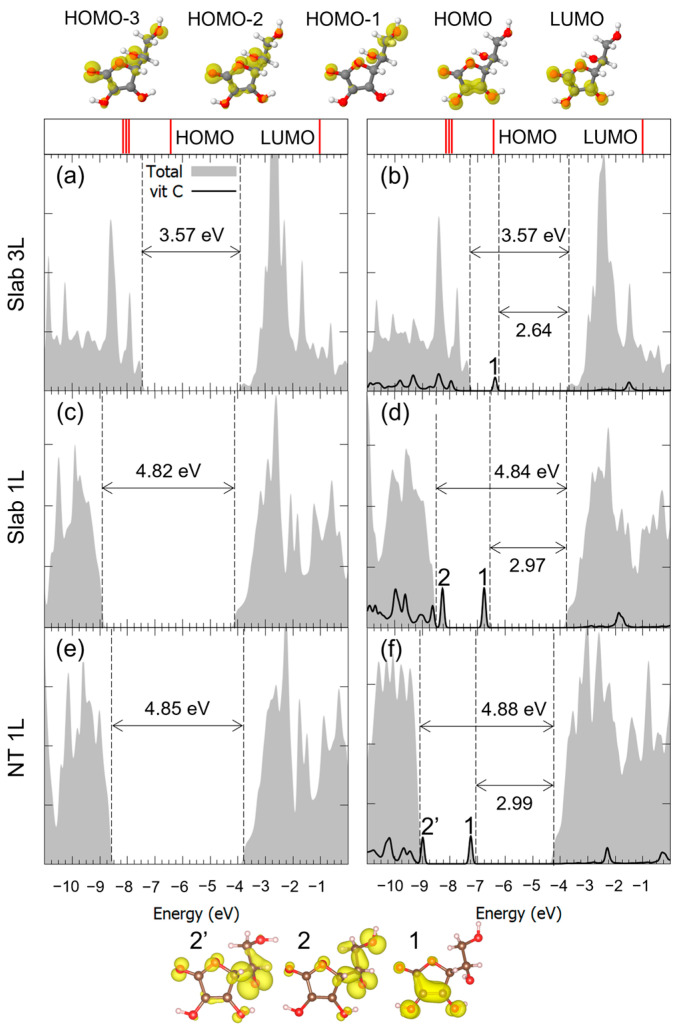
Total density of states (DOS) of TiO_2_ slab 3L (panels (**a**,**b**)), slab 1L (panels (**c**,**d**)) and NT 1L (panels (**e**,**f**)) before (left column) and after (right column) the adsorption of Vitamin C for the $M_{CO5}^{2Hbonds-D}$ adsorption mode. The projection on the atomic orbitals of Vitamin C is shown with a black line. The VB-CB gap and the HOMO-LUMO gap are marked by dashed lines. The zero energy is set to the vacuum level. In the upper part of the figure, the eigenvalues and charge densities of the molecular orbitals of an isolated Vitamin C molecule are represented using an isovalue of 0.001 e^−^/Bohr^3^. The states found within the TiO_2_ VB-CB gap are marked by numbers, and their related charge density plots are shown at the bottom of the figure with an isovalue of 0.0005 e^−^/Bohr^3^. C, H and O atoms are represented with dark gray, white and red spheres, respectively.

For the case of the M^2Hbonds-D^_CO5_ configuration on all three substrates, the density of states (DOS) is reported in Figure 5. We observed that, through comparison with the pristine flat or curved surfaces, the valence-band to conduction-band (VB-CB) energy gap was almost unchanged by the presence of the molecule. Also, in the case of the 1L surface, the electronic structure of the corresponding nanotube did not seem to be affected by the curvature of the TNT. Concerning Vitamin C, the DOS showed one molecular mid-gap state, which could be observed for all substrate thicknesses: both its absolute energy position and its charge density in real space allowed us to associate it with the almost unperturbed HOMO of the isolated Vitamin C molecule. At lower energies, the HOMO-n states were more hybridized with the TiO_2_ ones, while at higher energies, the LUMO partially retained its identity but seemed more hybridized than the HOMO.

**Figure 6:**
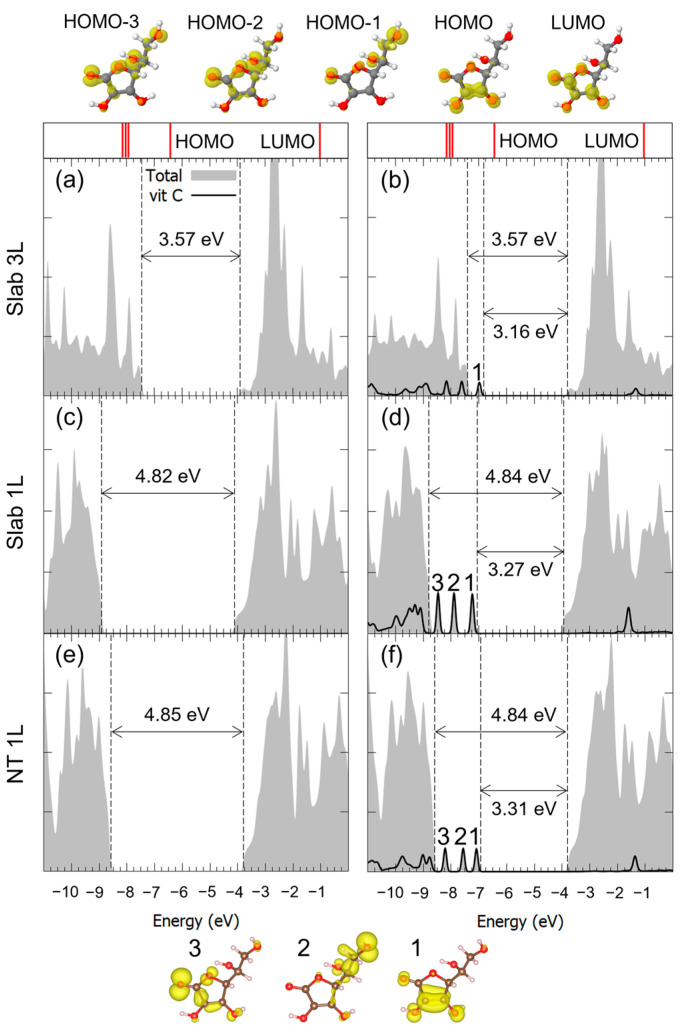
Total density of states (DOS) of TiO_2_ slab 3L (panels (**a**,**b**)), slab 1L (panels (**c**,**d**)) and NT 1L (panels (**e**,**f**)) before (left column) and after (right column) the adsorption of Vitamin C for the $M_{OHc2}^{2Hbonds-D}$ adsorption mode. The projection on the atomic orbitals of Vitamin C is shown with a black line. The TiO_2_ VB-CB gap and the HOMO-LUMO gap are marked by dashed lines. The zero energy is set to the vacuum level. In the upper part of the figure, the eigenvalues and charge densities of the molecular orbitals of an isolated Vitamin C molecule are represented using an isovalue of 0.001 e^−^/Bohr^3^. The states found within the TiO_2_ VB-CB gap are marked by numbers, and their related charge density plots are shown at the bottom of the figure using an isovalue of 0.0005 e^−^/Bohr^3^. C, H and O atoms are represented with dark gray, white and red spheres, respectively.

**Figure 7:**
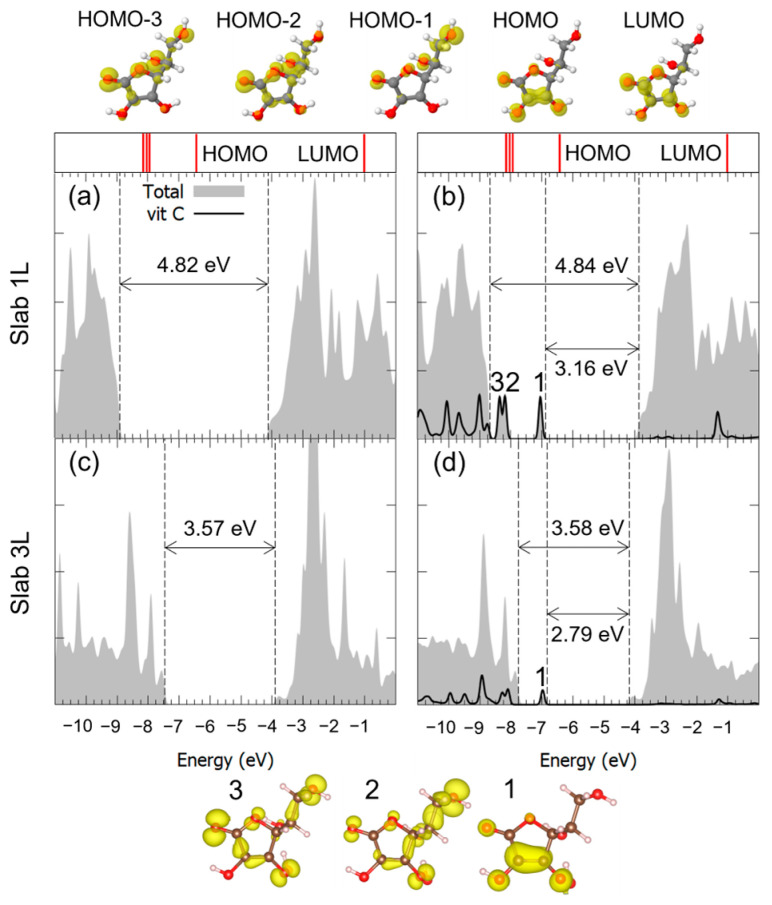
Total density of states (DOS) of TiO_2_ slab 1L (panels (**a**,**b**)) and slab 3L (panels (**c**,**d**)) before (left column) and after (right column) the adsorption of Vitamin C for the $B_{O(H)a3/OHa4}^{1Hbond-A}$ adsorption mode. The projection on the atomic orbitals of vitamin C is shown with a black line. The VB-CB gap and the HOMO-LUMO gap are marked by dashed lines. The zero energy is set to the vacuum level. In the upper part of the figure, the eigenvalues and charge densities of the molecular orbitals of an isolated Vitamin C molecule are represented using an isovalue of 0.001 e^−^/Bohr^3^. The states found within the TiO_2_ VB-CB gap are marked by numbers, and their related charge density plots are shown at the bottom of the figure with an isovalue of 0.0005 e^−^/Bohr^3^. C, H and O atoms are represented with dark gray, white and red spheres, respectively.

Similar considerations can be made for the adsorption of Vitamin C in the M^2Hbonds-D^_OHc2_ and to B^1Hbond-A^_O(H)a3/OHa4_ modes, whose DOSs are shown in Figure 6 and Figure 7. Both the VB-CB energy gap and the mid-gap states calculated in our model indicated the presence of an interaction between the molecule and the substrate, which was indeed bonding, as seen through the hybridization of some of the molecular orbitals. Because of the large diameter of the 3L TNT, and therefore its reduced curvature, the DOS for the Vitamin C adsorbed with the bidentate mode on such a substrate is not shown, given its similarity with the case of the 3L slab.

### 3.2. Vitamin C Adsorbed on the Anatase (001)A TiO_2_ Surface and the Corresponding Nanotube

**Figure 8:**
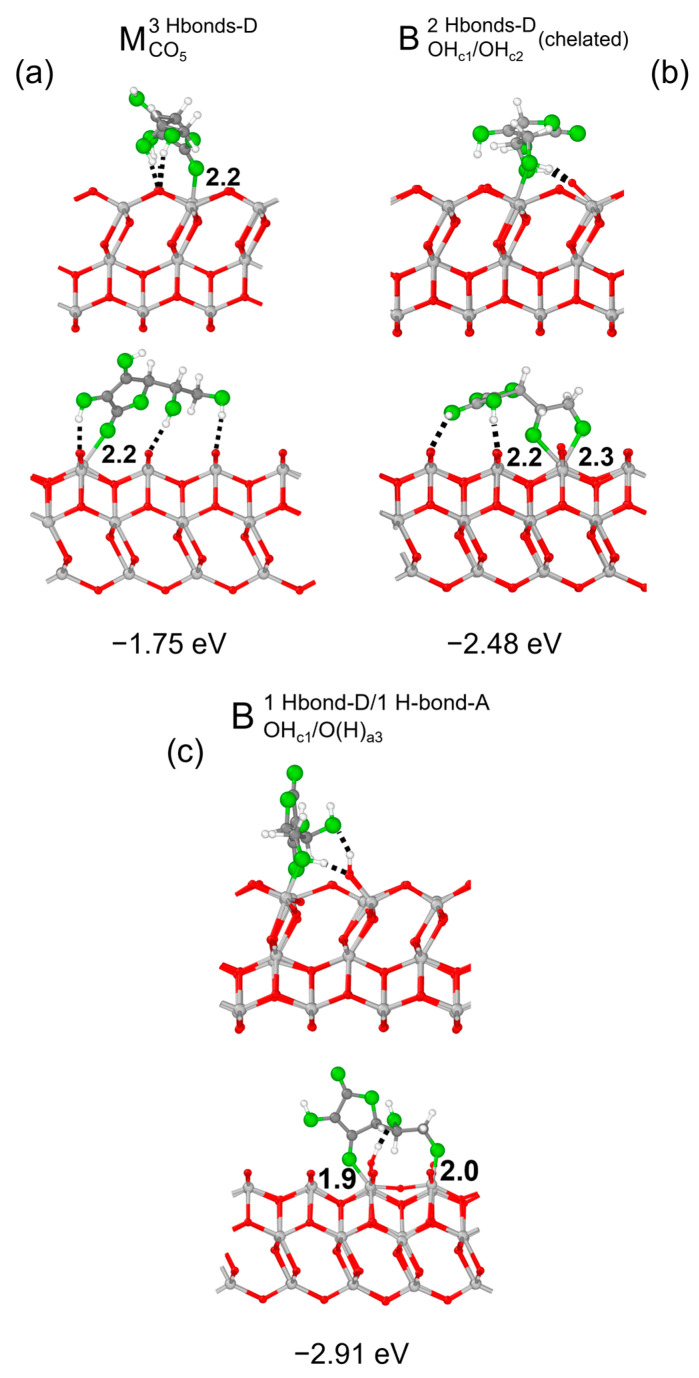
Adsorption configurations and energies per molecule (${\Delta E}_{\mathrm{ads}}^{\mathrm{mol}}$) for three optimized models of Vitamin C on the TiO_2_ (001)A 3L surface: $M_{CO5}^{3Hbonds-D}$ (panel **a**), $M_{OHc1/OHc2}^{2Hbonds-D}(\mathrm{chelated})$ (panel **b**) and $B_{OHc1/O(H)a3}^{1Hbond-D/1Hbond-A}$ (panel **c**). In each panel, the top/bottom image reports the front/side view along the [010]/[100] crystallographic direction. Ti, C and H atoms are represented with light gray, dark gray and white spheres, respectively. Oxygen atoms of Vitamin C/slab are represented with green/red spheres. Solid and dashed black lines mark the electrostatic and hydrogen bonds, respectively. Relevant distances are reported in Å.

We now investigate the adsorption of Vitamin C on the more reactive (001)A surface, and we begin by surveying of the possible adsorption configurations. The resulting optimized structures are shown in Figure 8: as expected, the overall adsorption intensity was stronger than for the (101) surface (compare with Figure 2b,d,f). The only anomalous result was for the monodentate (M^3Hbonds-D^_CO5_, Figure 8a) configuration, which had a similar structure to that on the 3L (101) surface of Figure 2d, but with a weaker adsorption, because the hydroxyl group at the end of the side chain formed an additional H-bond on the (001)A surface rather than interacting electrostatically with the substrate. The two bidentate configurations, instead, were rather different than that reported for the (101) surface. First of all, the functional groups involved in the bonds were different: on the (101) surface, two undercoordinated surface Ti atoms interacted with both the hydroxyl groups of the C ring of the molecule, whereas on the (001)A surface, Vitamin C could bind either through both the hydroxyl groups of the side chain in a chelated fashion (B^2Hbonds-D^_OHc1/OHc2_, Figure 8b) or one OH group of the C ring and the last one of the side chain (B^1Hbonds-D/1Hbonds-A^_OHc1/O(H)a3_, Figure 7c). Interestingly, these two configurations on the (001)A surface show an additional interaction mechanism, related to the structure of this surface: the undercoordinated Ti atoms on the surface which were bound with the molecule broke one bond with a surface-bridging O atom, which became prone to form an H-bond with the same OH groups bonding with the Ti atom.

Next, we focused our attention on the B^1Hbonds-D/1Hbonds-A^_OHc1/O(H)a3_ structure, and we rolled up the surface into the nanotube with the molecules placed in the inner zone, with its long axis aligned to the axis of the TNT: this configuration was chosen in order to reproduce the same structure of the nanotube that has been investigated and reported by Ferrari and co-workers [34] as the only one surface with negative strain energy. 

**Figure 9:**
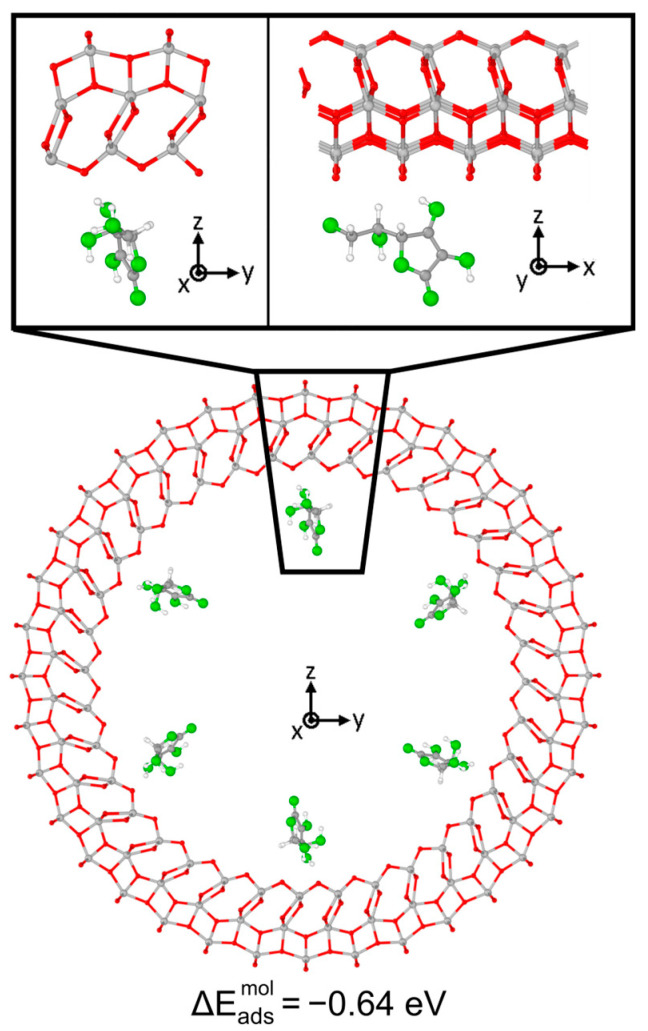
Adsorption configuration and energy for Vitamin C placed inside the TiO_2_ NT (001)A 3L (36, 0). In the inset, the top/bottom image shows a zoom-in of the side/front view. Ti, C and H atoms are represented with light gray, dark gray and white spheres, respectively. Oxygen atoms of Vitamin C/slab are represented with green/red spheres. Solid and dashed black lines mark the electrostatic and hydrogen bonds, respectively. Relevant distances are reported in Å.

**Figure 10:**
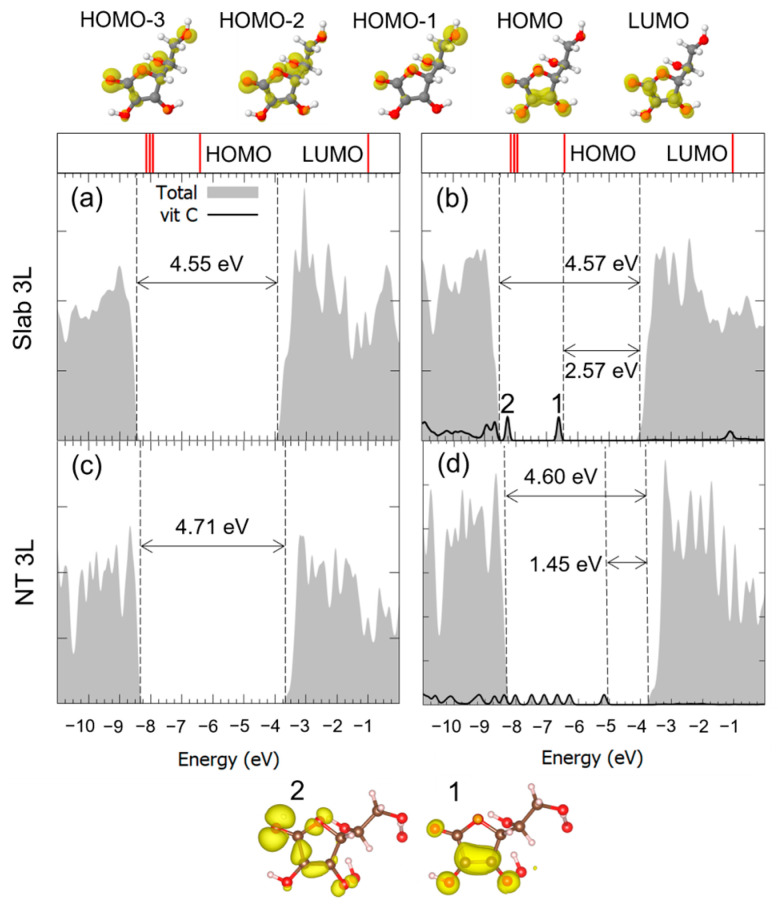
Total density of states (DOS) of TiO_2_ slab 3L (panels (**a**,**b**)) and NT 3L (panels (**c**,**d**)) before (left column) and after (right column) the adsorption of Vitamin C for the $B_{\mathrm{OHc}1/O(H)a3}^{1Hbond-D/1Hbond-A}$ adsorption mode. The projection on the atomic orbitals of vitamin C is shown with a black line. The VB-CB gap and the HOMO-LUMO gap are marked by dashed lines. The zero energy is set to the vacuum level. In the upper part of the figure, the eigenvalues and charge densities of the molecular orbitals of an isolated Vitamin C molecule are represented using an isovalue of 0.001 e^−^/Bohr^3^. The states found within the TiO_2_ VB-CB gap are marked by numbers, and their related charge density plots are shown at the bottom of the figure with an isovalue of 0.0005 e^−^/Bohr^3^. C, H and O atoms are represented with dark gray, white and red spheres, respectively.

The optimized geometry is shown in Figure 9: the adsorption of Vitamin C on the nanotube was notably weaker than that on the corresponding 3L slab, as the absolute value of the adsorption energy decreased from −2.91 eV to −0.64 eV. No chemical bonds and proton transfers were observed with the substrate, only weak physical interactions. This different behavior of the molecule is reflected by the electronic properties of the system: the comparison of DOS of the slab with that of the nanotube, before and after the adsorption, is reported in Figure 10. In the case of the curved substrate, a much larger number of mid-gap states could be observed in the VB-CB gap of the TiO_2_, which were almost unperturbed molecular orbitals. 

Since the only difference with the B^1Hbonds-D/1Hbonds-A^_OHc1/O(H)a3_ configuration on the 3L slab is the curvature of the substrate and the consequent structural distortion of the internal top layer atoms, we relate this different binding mode of the Vitamin C on the nanotube to a resulting unfavorable configuration of the exposed Ti atoms, which prevented the overlap of the Ti 3d orbitals and the OH groups of the molecule. This mismatch was more evident for the (001)A surface than for the (101) one, where a similar weaker interaction was observed, supporting our hypothesis that the adsorption of the Vitamin C is driven by geometrical factors.

## 4. Conclusions

In this work, we investigated the adsorption of Vitamin C molecules on nanotube models derived from both the anatase (101) and (001)A TiO_2_ surfaces. We carried out a comparative analysis through hybrid density functional theory (B3LYP) calculations of several adsorption configurations, as we considered, besides different anatase surfaces, different rolling directions of the nanotube and different orientations and positions of the molecule with respect to the nanotube (e.g., inside or outside). We identified the most important trends in the adsorption properties, also taking into account the electronic properties of the system, through comparison with corresponding flat surface models. We exploited our observations on the thinner slab systems in order to obtain additional insights into thicker and more realistic models of nanotubes.

Our calculations suggest that the interaction between the Vitamin C and TiO_2_ nanotubes, derived by both the most stable (101) and the more reactive (001)A surfaces, is driven by geometrical factors, i.e., the correct disposition and orientation for interaction between the undercoordinated Ti atoms on the surface and any of the functional groups of the molecule. The thickness of the TNT is a crucial parameter, since for thinner (1L) nanotubes, the adsorption strength is similar to that of the corresponding flat slab, whereas for thicker (3L) nanotubes, the interaction is substantially weaker. On the contrary, we must note that the rolling up direction of the nanotube and the alignment of its axis with that of the molecule have a smaller impact on the drug adsorption properties. 

The present study has focused on the adsorption modes and relative stability of Vitamin C on different flat or curved surfaces. The binding energy values that have been obtained on large nanotubes (after BSSE correction) are compatible with a drug release mechanism based on pH variation from physiological to acidic, where Vitamin C is expected to be undissociated and therefore only weakly physisorbed on the walls of large nanotubes. However, further details on the drug release mechanism will be the object of a future study, where solvent and ionic strength must also be taken into account.

## Data Availability

Data are contained within the article.

## Supplementary Materials

The following supporting information can be downloaded at: https://www.mdpi.com/article/10.3390/nano14030261/s1, Figure S1: structure of TiO_2_ (001), (001)A, (001)B surfaces; Figure S2: structure of TiO_2_ (101) 3L nanotubes with different radius and slab; Figure S3: structure of TiO_2_ (001)A 3L nanotubes with different radius and slab; Figure S4: structure and adsorption energy of additional Vitamin C models on TiO_2_ (101) 1L flat surface; Figure S5: optimized B^1Hbond-A^_O(H)a3/OHa4_ model of Vitamin C adsorbed on TiO_2_ (101) 1L nanotubes.
