# Supplementary material for: Vitamin C Affinity to TiO2 Nanotubes: A Computational Study by Hybrid Density Functional Theory Calculations
Source: Nanomaterials (Basel). 2024 Jan 25;14(3):261. doi: 10.3390/nano14030261 (PMC10856687; doi:10.3390/nano14030261)
Supplement: Supplementary file 1 [file nanomaterials-14-00261-s001.zip › nanomaterials-2795720-supplementary.pdf]

SUPPLEMENTARY MATERIALS for:

Vitamin C Affinity to TiO_2 Nanotubes: A Computational Study by Hybrid Density Functional Theory Calculations

Aldo Ugolotti,[a] Mirko Dolce,[a] Cristiana Di Valentin[a,b,*]

[a] Dipartimento di Scienza dei Materiali, Università di Milano-Bicocca, via R. Cozzi 55, 20125 Milano, Italy

[b] BioNanoMedicine Center NANOMIB, Università di Milano-Bicocca, via R. Follereau 3, 20854 Vedano al Lambro, Italy

[*] Corresponding author: cristiana.divalentin@unimib.it

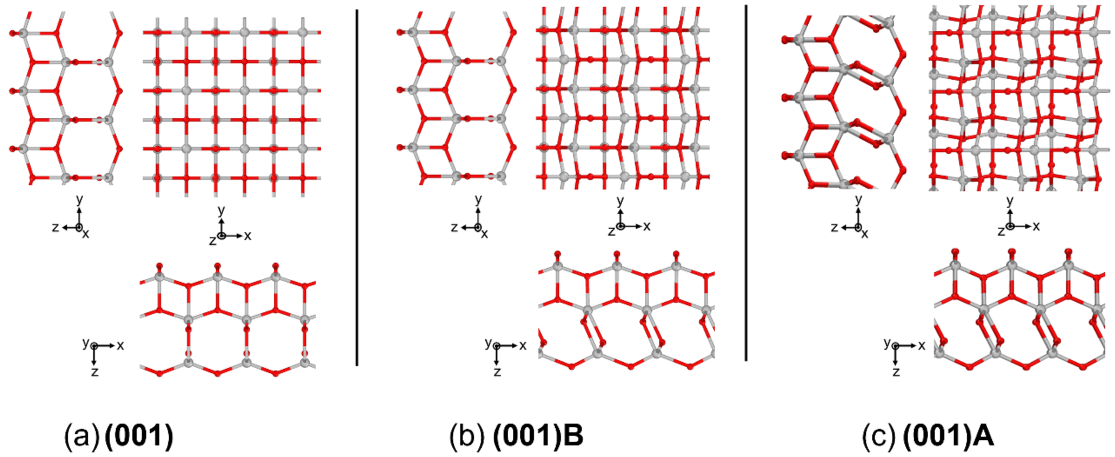

Figure S1: Structure of TiO_2 3L slabs for the (001) (panel a), (001)B (panel b) and (001)A (panel c) surfaces. Surface (001)B can be constructed through a rigid shift of the highest triatomic layer along the x direction. Surface (001)A is obtained with an additional shift of the lowest triatomic layer along the –y direction [Ref.: Ferrari et al., J. Phys. Chem. Lett. 1, 19, 2854–2857 (2010)]. Each panel reports a top view and the side views along [010] and [100] crystallographic directions. Ti and O atoms are represented with light grey and red spheres, respectively.

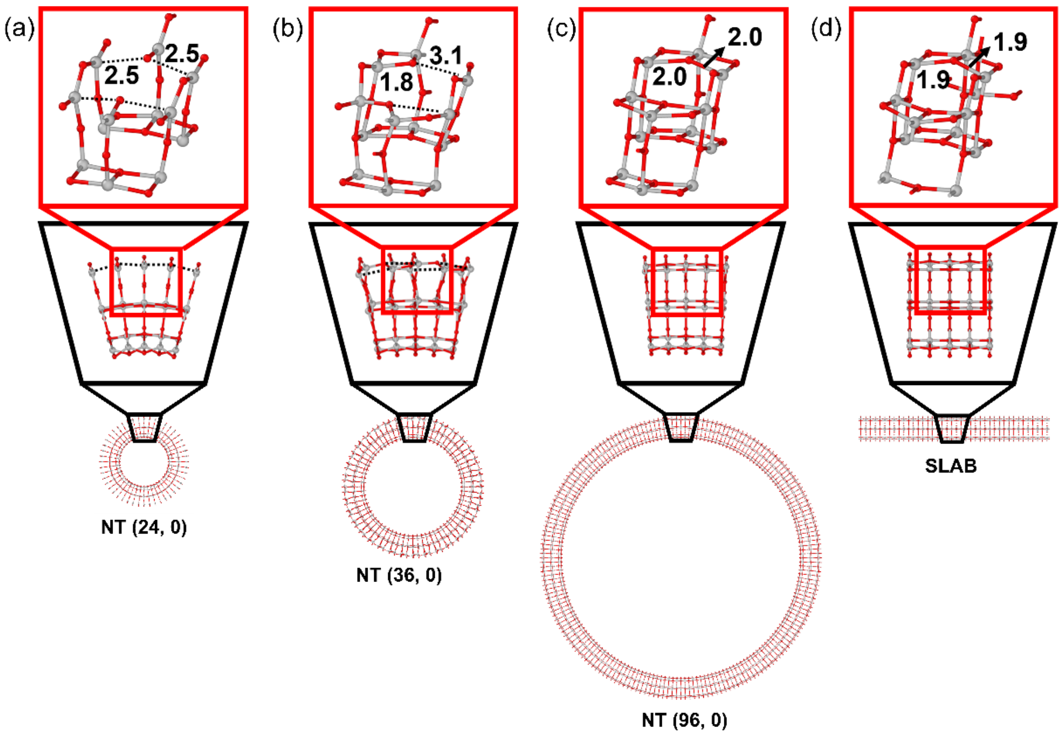

Figure S2: Structure of TiO_2 NT (101) 3L with different diameters and of the related flat (101) surface. Each panel includes two different views of the structure; dashed black lines mark the broken bonds. Ti and O atoms are represented with light grey and red spheres, respectively. Relevant distances are reported in Å.

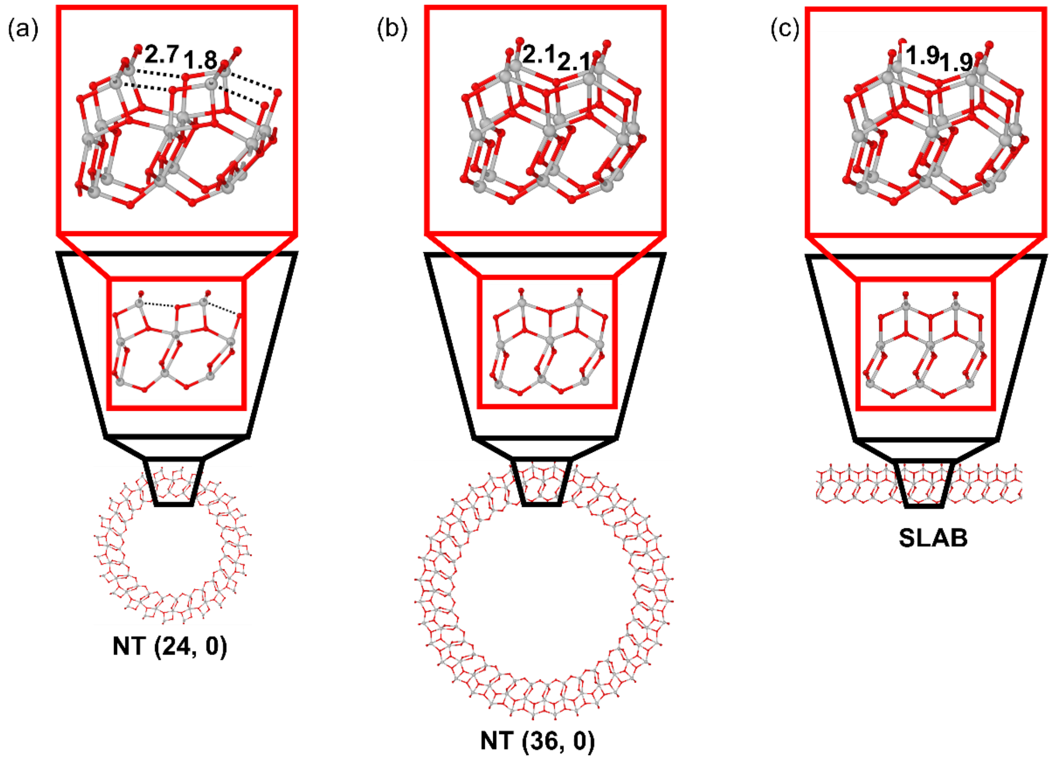

Figure S3: Structure of TiO_2 NT (001)A 3L with different diameters and of the related flat (001)A surface. Each panel includes two different views of the structure; dashed black lines mark the broken bonds. Ti and O atoms are represented with light grey and red spheres, respectively. Relevant distances are reported in Å.

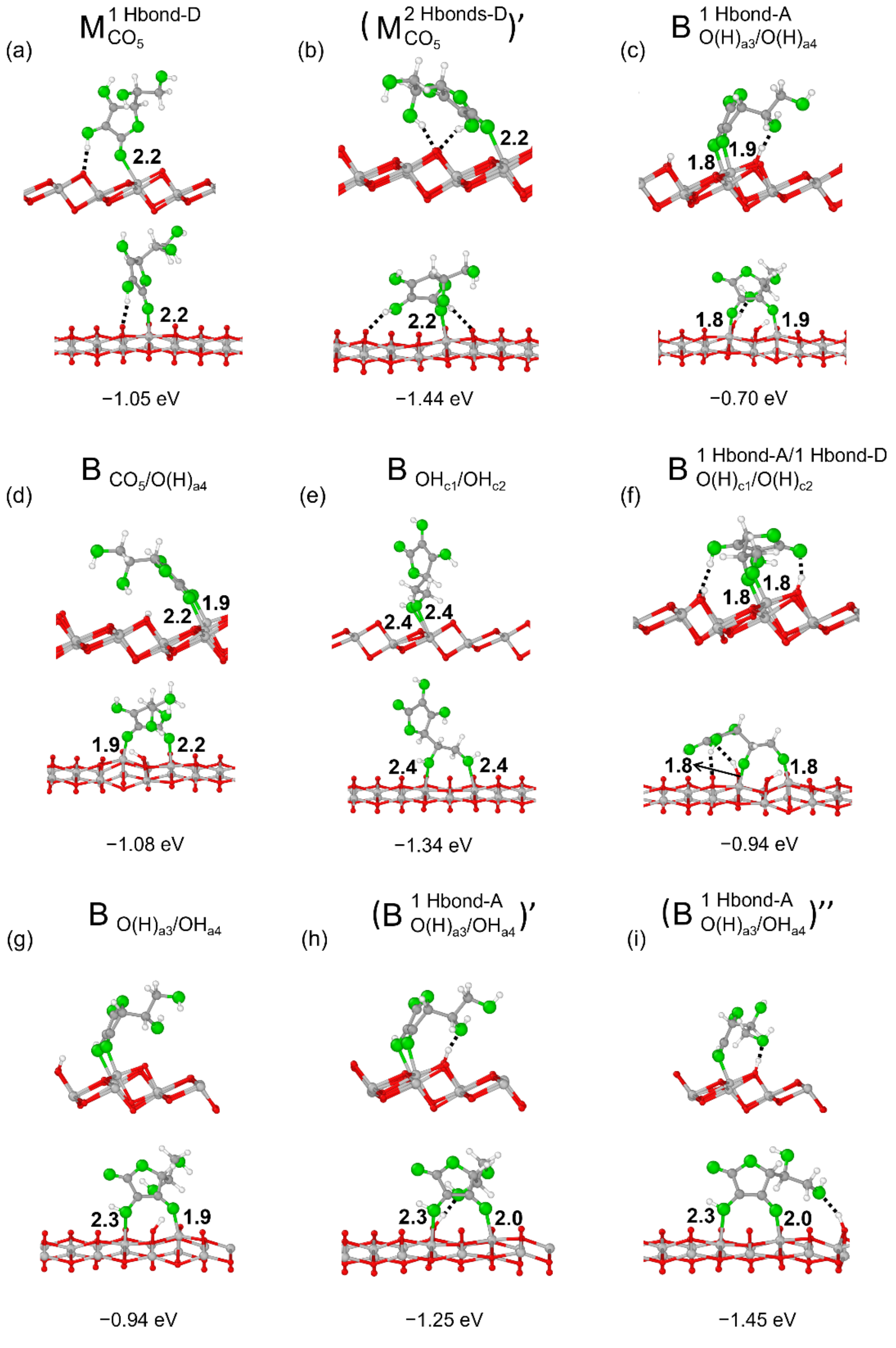

Figure S4: Adsorption configurations and energies per molecule (ΔE_{ads}^{mol}) of additional optimized models of Vitamin C on the TiO_2 (101) 1L surface (panels a-i). Panels g, h and i report models that differ in the position of the transferred proton. In each panel, the top/bottom image reports the side view along the [010]/[10-1] crystallographic direction. Ti, C, H atoms are represented with light grey, dark grey and white spheres, respectively. Oxygen atoms of Vitamin C/slab are represented with green/red spheres. Solid and dashed black lines mark the electrostatic and hydrogen bonds, respectively. Relevant distances are reported in Å.

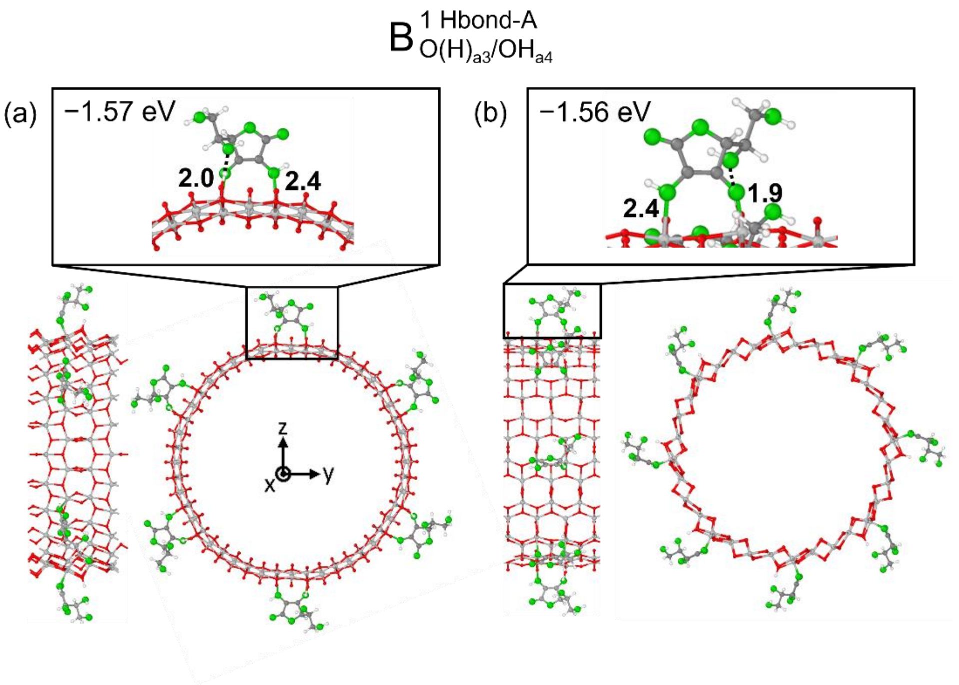

Figure S5: Optimized configurations and adsorption energies per molecule (ΔE_{ads}^{mol}) for a single adsorption mode of Vitamin C on different TiO_2 NT (101) 1L configurations. In panel a the NT is constructed with a (24, 0) roll-up index while in panel b with a (0, 24) index.

In each panel, the left/right image reports the front/side view while on top a zoom on the molecule is shown. Ti, C, H atoms are represented with light grey, dark grey and white spheres, respectively. Oxygen atoms of vitamin C/slab are represented with green/red spheres. Solid and dashed black lines mark the electrostatic and hydrogen bonds, respectively. Relevant distances are reported in Å.
